# Predictors of Inpatient Utilization among Veterans with Dementia

**DOI:** 10.1155/2014/861613

**Published:** 2014-05-29

**Authors:** Kyler M. Godwin, Robert O. Morgan, Annette Walder, David M. Bass, Katherine S. Judge, Nancy Wilson, A. Lynn Snow, Mark E. Kunik

**Affiliations:** ^1^Houston VA HSR&D Center for Innovations in Quality, Effectiveness and Safety, Michael E. DeBakey VA Medical Center, (MEDVAMC 152), 2002 Holcombe Boulevard, Houston, TX 77030, USA; ^2^Baylor College of Medicine, One Baylor Plaza, Houston, TX 77030, USA; ^3^Memorial Hermann Hospital, Houston, 6411 Fannin, TX 77030, USA; ^4^The University of Texas School of Public Health, 1200 Herman Pressler, Rm. E-343, Houston, TX 77030, USA; ^5^Margaret Blenkner Research Institute, Benjamin Rose Institute on Aging, 11890 Fairhill Road, Cleveland, OH 44120, USA; ^6^Cleveland State University, 2121 Euclid Avenue, Cleveland, OH 44115, USA; ^7^Center for Mental Health and Aging and Department of Psychology, The University of Alabama, P.O. Box 870315, Tuscaloosa, AL 35487, USA; ^8^Tuscaloosa VA Medical Center, 3701 Loop Road, Tuscaloosa, AL 35404, USA; ^9^VA South Central Mental Illness Research, Education and Clinical Center (A Virtual Center), USA

## Abstract

Dementia is prevalent and costly, yet the predictors of inpatient hospitalization are not well understood. Logistic and negative binomial regressions were used to identify predictors of inpatient hospital utilization and the frequency of inpatient hospital utilization, respectively, among veterans. Variables significant at the *P* < 0.15 level were subsequently analyzed in a multivariate regression. This study of veterans with a diagnosis of dementia (*n* = 296) and their caregivers found marital status to predict hospitalization in the multivariate logistic model (*B* = 0.493, *P* = 0.029) and personal-care dependency to predict hospitalization and readmission in the multivariate logistic model and the multivariate negative binomial model (*B* = 1.048, *P* = 0.007, *B* = 0.040, and *P* = 0.035, resp.). Persons with dementia with personal-care dependency and spousal caregivers have more inpatient admissions; appropriate care environments should receive special care to reduce hospitalization. This study was part of a larger clinical trial; this trial is registered with ClinicalTrials.gov NCT00291161.

## 1. Introduction

Dementia is a prevalent and costly disease. In 2012, approximately 5.2 million Americans aged 65 and older had dementia, which accounted for $200 billion in healthcare payments [1]. By 2050, these numbers are projected to increase to a staggering 11–16 million persons with dementia (PWD) and $1.1 trillion in payments for care [1]. This high cost of care can be attributed to increased utilization of healthcare resources among PWD, compared with older adults without dementia [2–5]. Specifically, the number of inpatient hospital stays [1] and home-health and nursing-home use is higher for PWD [5] than for older adults without dementia.

Because of the large cohort of older adults, the prevalence and cost of dementia have significant implications for healthcare resources, both currently and in the future. Dementia-related healthcare utilization is a significant problem for both public and private healthcare systems. Veterans' Affairs (VA) is the largest single-payer healthcare system in the United States. The prevalence of dementia among veterans 65 years of age and older in the VA is 7.3%, comparable to the prevalence of dementia among males in the civilian population reported in the literature [6]. As in the general population, this number is expected to rise with the aging of the current population. Additionally, similar to PWD in the general population, veterans with dementia have demonstrated higher rates of inpatient hospital utilization and longer inpatient stays than veterans without dementia [6].

Predictors of healthcare utilization have been identified in veteran and older-adult populations. Specifically, among veterans who had sustained a traumatic brain injury, increased age and disability were significant predictors of hospitalization [7]. Among veterans with coexisting depression and dementia, inpatient utilization was significantly higher than for veterans with either depression or dementia [8]. Likewise, among persons aged 75 and older in Switzerland, depression was a significant predictor of readmission to an inpatient hospital, nursing-home admission, and death [9]. Additionally, physical disability, lower income, and lack of alternative healthcare options have also been associated with increased inpatient utilization among a sample of veterans who were, on average, over 60 years of age [10].

The Behavioral Model of Health Service Utilization [11] is one of the most commonly used models of healthcare utilization, incorporating both individual and contextual determinants of health into one model. Three major components, predisposing characteristics, enabling characteristics, and need factors, can predict healthcare utilization. Predisposing characteristics are considered to be the demographic and social dimensions of a community, such as age, gender, marital status, ethnicity, and educational level [12]. Enabling characteristics are financial resources that may be available to pay for healthcare services as well as the resources to travel to needed services and the time required to wait for such services [12]. Need characteristics include health-related characteristics of the environment (i.e., air and water quality), diagnosis of illness or disease, objective measurements of health status (i.e., blood pressure, weight, and temperature), and an individual's perception of his/her physical, emotional, and functional health status [12].

Due to the functional limitations resulting from dementia, most PWD who remain in the community have a caregiver, often a spouse or another family member, to provide day-to-day care and assistance. The influence that the caregiver has over the PWD's access to healthcare services can be conceptualized as a contextual determinant of health in the Behavioral Model of Health Service Utilization. Specifically, a caregiver's perception of his or her emotional, physical, and functional health status has been classified as a “need characteristic” in the Behavioral Model of Health Service Utilization [13].

Despite the prevalence of and high costs associated with dementia, few studies have examined the predictors of inpatient utilization among PWD [14, 15]. The veteran population is appropriate for such study because of its similarities to the civilian population in terms of prevalence and utilization [6]. Additionally, because the VA is a single-payer system, the electronic health record facilitates ready measurement of utilization of inpatient healthcare services. Elucidation of the predictors of inpatient hospitalization may provide opportunities for early intervention to reduce the rate of hospitalization among PWD. Thus, this study sought to determine the predictors of inpatient hospitalization, based on the Behavioral Model of Health Service Utilization, by examining a population of veterans with dementia.

## 2. Materials and Methods

This study is a secondary data analysis of a previously completed study of PWD and their caregivers called Partners in Dementia Care (PDC). PDC was a telephone-based, care-coordination intervention for veterans with dementia and their family caregivers. It was unique in the fact that it relied on a formal partnership between local VA medical centers and local chapters of the Alzheimer's Association to link veterans and their caregivers to needed resources.

**Figure 1:**
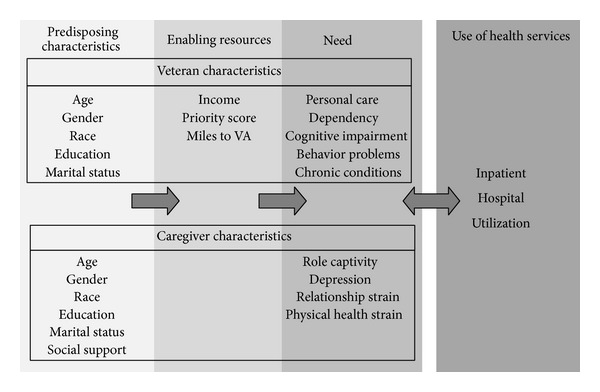
Behavioral model of inpatient hospitalization for veterans with dementia.

The Behavioral Model of Health Service Utilization—with its major components of predisposing characteristics, enabling resources, and clinical need—guides this examination of predictors of inpatient hospital utilization among veterans with dementia (see Figure 1). Specifically, inpatient hospital utilization was the primary outcome of this study. Sociodemographic variables were considered as predisposing variables. Enabling resources included the veteran's income, priority score, and miles from his/her home to the VA. Clinical-need variables included the veteran's self-rated health, personal-care dependency, cognitive impairment, behavior problems, and chronic conditions, as well as the caregiver's role captivity, depression, relationship strain, and physical health strain.

### 2.1. Recruitment and Data Collection

Veterans and their caregivers were recruited from five VA facilities (Boston, MA; Houston, TX; Providence, RI; Oklahoma City, OK; and Beaumont, TX). The study was conducted throughout these five VA facilities in partnership with their local chapters of the Alzheimer's Association. The Houston and Boston locations received the intervention, while the Providence, Oklahoma City, and Beaumont locations served as the controls. All participants received educational material about dementia at the beginning of the study. Study participants gave their informed consent to participate, and the study protocol was approved by the institutional review board of Baylor College of Medicine and affiliated hospitals.

Participants were recruited in one of two ways. Veterans with a recent diagnosis of dementia and their family caregivers were directly referred to the parent study by their VA physician. Additionally, searching the VA electronic medical records identified veterans with a dementia diagnosis (eligible International Classification of Diseases, Ninth Revision, diagnostic codes were 290.41-43, 291.2, 292.82, 294.1, 294.8, and 331.0) in the prior two years. These veterans and their family caregivers were sent a letter to assess interest in the study. A trained research assistant followed-up with them one week later to explain the study in detail, screen them for eligibility, answer questions, and obtain verbal informed consent. After obtaining the signed informed consent, trained research staff collected data from patients and their caregivers over the phone during three different interviews over a one-year period. Data were collected after recruitment (baseline) and at 6 and 12 months after beginning the study.

### 2.2. Sample

Veterans who were at least 50 years old, received their primary care at the VA, resided within the Alzheimer's Association local chapter's service area, and had a documented diagnosis of dementia in their medical record were recruited for participation in PDC. Caregivers of veterans were simultaneously recruited. While most veterans had caregivers, having a caregiver was not one of the eligibility criteria. Caregivers had to be a family member or friend of the veteran and had to provide assistance with activities of daily living, including personal care and health-related decisions.

### 2.3. Measures

All measures, with the exception of the outcome measure, were collected at baseline. Sociodemographic information, including age, gender, race, education, marital status, and income, were collected from the caregiver for both the patient and the caregiver. Each veteran's priority score was collected from VA administrative data. A veteran's priority score is based on his/her service and/or service-connected disability and determines the extent of his/her healthcare coverage within the VA medical system. Priority scores range from 1 to 8b, with 1 being the highest priority for enrollment. For this study, veterans were grouped into three sets of priority levels (i.e., 1, 2–6, and 7a–8b), which broadly differentiated copayment levels and out-of-pocket maximums [16]. The distance the veteran lived from the VA was calculated in miles, based on the veteran's address.

Data about the veterans' health and functional status were collected from the PWD when they were able to answer questions via telephone. If the PWD was unable to provide the information, the data were collected from the caregiver. Caregivers were not asked to validate the responses of the PWD. Veterans' health status was measured with self-reported data and VA administrative records, as well as validated questionnaires. The patients' chronic conditions other than dementia were obtained from both asking the caregiver and querying VA medical records. Patients' personal-care dependency is the sum of six items (answered on a three-point scale of “no difficulty” to “a great deal of difficulty”), assessed by asking the patient and caregiver about the number of dependencies the patient had in personal care (i.e., toileting, bathing, grooming, dressing, eating, and mobility) and instrumental activities of daily living (i.e., managing finances, scheduling appointments, or taking care of the home). It has good reliability, with a Cronbach's *α* of 0.87 [17]. Veterans' cognitive impairment was assessed by summing seven items, answered on a three-point scale (0, 1, 2), that ask about difficulties with memory, such as knowing the day of the week, keeping track of current events, paying attention, repetitive verbalizations, and remembering persons, places, and appointments. Total scores range from 0 to 14, with higher scores indicating greater cognitive impairment. This measure has a Cronbach's *α* of 0.82 [17]. Problem behaviors were assessed by a four-item, previously validated survey that asked about the frequency (“none of the time,” “some of the time,” or “most or all of the time”) of neuropsychiatric symptoms (i.e., yelling or swearing, complaining or criticizing, interfering with family members, and agitation). Total scores ranged from 0 to 12; higher scores indicated a greater frequency of problem behaviors. This measure has a Cronbach's *α* of 0.79 [17].

The effect that caregiving had on the caregiver was measured with previously tested, valid, and reliable questionnaires. Symptoms of depression were assessed with the 10-item Center for Epidemiologic Studies Depression Scale (CES-D) [18]. The CES-D had a Cronbach's *α* of 0.78 in this study [17]. Role captivity, or how trapped the person feels because of his/her caregiving role [19], was assessed with a three-item composite measure and had a Cronbach's *α* of 0.80 [20]. Relationship strain was measured with a previously validated, six-item scale that included “Do you feel closer to the veteran?,” “Do you feel appreciated by the veteran?,” “Do you get pleasure from helping?,” “Are you angry towards the veteran?,” “Do you feel the relationship is strained?,” and “Do you feel manipulated by the veteran?.” These six items are summed for a score of 0–18, where higher scores indicate great strain on the relationship because of the caregiving role. It had a Cronbach's *α* of 0.78 [17]. Physical health strain was assessed with a previously validated, three-item scale that asked if, because of caregiving, a caregiver's physical health is worse, if she/he gets sick more often, or she/he experiences more aches and pains. The three items are summed to a total score ranging from 0 to 9. Higher scores indicate greater strain. Physical health strain had a Cronbach's *α* of 0.83 [17]. Additionally, caregivers were also asked to report on the number of family and friends who were available to help care for the PWD.

Two methods were used to measure the primary outcome of inpatient hospitalization. VA medical records were obtained to verify all VA inpatient hospital admissions. Additionally, veterans and their caregivers self-reported any inpatient hospitalization outside the VA. Data regarding inpatient hospitalization were collected over the 12 months of the study, with inpatient hospitalization defined both dichotomously (i.e., if the veteran had any inpatient hospitalization during the 12 months of the study) and numerically (i.e., the total number of inpatient admissions that a veteran had during the 12 months of the study).

### 2.4. Data Analysis

Descriptive statistics were calculated for all study variables. The number of inpatient hospitalizations over time was grouped into three categories: “baseline,” “6 months,” and “12 months.” “Baseline” was from 1 year prior to recruitment through the first assessment. “6 months” was from after the first assessment (baseline) to 6 months. “12 months” was from after the second assessment (6 months) to 12 months.

Logistic regression models were used to assess whether individual predisposing, enabling, and need factors predicted any inpatient hospital utilization (“yes” or “no”). Negative binomial regression was used to assess whether predisposing, enabling, and need factors predicted the frequency of inpatient hospital utilization among those veterans who had at least one inpatient admission. Both logistic and negative binomial regressions controlled for site by including site as a variable in all of the models. Marital status and caregiver variables (i.e., depression, role captivity, relationship, and physical health strain) were also included in the models. Level of significance was set at the *P* < 0.15 level to include the broadest set of predictors in the multivariate models.

Predisposing, enabling, and need variables that were found to be significant at the *P* < 0.15 level in the univariate logistic and univariate negative binomial regressions were then included together in the multivariate logistic and multivariate negative binomial regressions. The multivariate regressions were also controlled for by site. Statistical significance was assessed at the *P* < 0.05 level for the multivariate analyses.

## 3. Results

### 3.1. Sample Characteristics

The sample consisted of 296 veterans with a diagnosis of dementia and their caregivers. Average age for veterans and caregivers was 78.6 and 68.8 years, respectively (Table 1). Most veterans were married, white, non-Hispanic men. More than half of veterans and caregivers had greater than a high school education. Approximately 40% of veterans had an income between $20,000 and $40,000 and lived at an average of 20 miles from their VA. Most veterans had a priority score of 2–6, indicating they were in the midrange of healthcare benefit eligibility.

Approximately 35% (*n* = 104) of veterans had an inpatient hospital admission during the study (see Table 2, which shows the number of veterans with inpatient hospital admissions over 12 months). The majority of inpatient admissions were in non-VA hospitals. The mean number of total inpatient admissions increased over the year, with participants averaging an increase of 2.87 inpatient admissions between the 6- and 12-month periods. Veterans with dementia who had an inpatient hospitalization had significantly higher scores on personal-care dependency (*P* = 0.007).

### 3.2. Predictors of Utilization

PWD's marital status (*B* = 0.502, *P* = 0.031) and personal-care dependency (*B* = 1.108, *P* = 0.007) were found to be significant predictors of inpatient hospitalization in the univariate logistic models (Tables 3 and 4). PWD's behavioral problems (*B* = 0.053, *P* = 0.058) and personal-care dependency (*B* = 0.05, *P* = 0.014) were significant in the univariate negative binomial models (Table 4). None of the enabling factors (income, veteran priority score, or miles the PWD lived from the VA) was found to be significant in either the logistic or negative binomial models (Table 5). When examined in a multivariate analysis, only marital status (*B* = 0.493, *P* = 0.029) and personal-care dependency (*B* = 1.048, *P* = 0.007) remained significant in the logistic model; only personal-care dependency (*B* = 0.04, *P* = 0.035) remained significant in the negative binomial model (Table 6). Thus, any inpatient hospital use was more likely by veterans who were married and had high personal-care dependency rather than by those who were single with low personal-care dependency. Further, increased frequency of inpatient hospital use among veterans with any use was more likely by veterans with high personal-care dependency rather than by those with low dependency.

## 4. Discussion

This study found that approximately one-third of veterans with dementia had an inpatient hospital admission over the course of one year, with the number of average admissions increasing during the year. Unlike previous studies [7, 10, 21, 22], age, gender, and ethnicity did not predict utilization. The only predisposing factor to predict inpatient utilization was marital status; married veterans were significantly more likely to have at least one hospital admission. None of the enabling factors selected for this study (income, priority score, and miles to the VA) was related to the likelihood of inpatient admissions.

Of the need factors examined, only personal-care dependency predicted an increased likelihood of a hospitalization. In the univariate analysis, exhibiting a high number of behavioral problems by veterans also predicted hospitalizations. However, this association was not maintained in the multivariate analysis, suggesting that personal-care dependency was the main explanatory factor. Veterans with dementia who had high personal-care dependency were more likely to have both an initial hospital admission and a hospital readmission than veterans with dementia with lower personal-care dependency. None of the caregiver-related need factors was found to be predictive of inpatient utilization.

The finding that high personal-care dependency, a measure of the veteran's functional dependence, predicted inpatient utilization indicates that a PWD's physical or cognitive limitations and/or his/her behavioral problems lead to increased hospitalization. This is congruent with the finding of other studies that functional limitations are predictive of inpatient utilization in other populations. Additionally, hospital admission has been associated with self-reported physical health [10] as well as with disability ratings among veterans with traumatic brain injury [7]. Additionally, because of dementia's effects on cognition, function, language, and perception, the PWD's level of dependency is increased, his/her ability to self-manage chronic conditions is decreased, and his/her ability to convey these challenges to the caregiver or medical provider may be impaired [23], possibly resulting in delayed primary care and increased hospitalization [24]. For example, Phelan and colleagues [24] found that hospitalization for potentially preventable admissions, such as bacterial pneumonia, congestive heart failure, and urinary-tract infections, was higher among PWD than among persons without dementia. Similarly, Bynum et al. found that ambulatory care-sensitive hospitalizations were greater among PWD [14], while Thorpe and colleagues found that, among veterans, the likelihood of ambulatory care-sensitive hospitalizations was greater among those living in rural areas than among those living in large metropolitan areas [15], possibly because of the challenge of obtaining timely and effective outpatient care in rural areas.

Although not surprising that personal-care dependencies contribute to hospital admissions and readmissions, there is a need to develop interventions to prevent both admissions and readmissions. Additional caregiver training around problems encountered among PWD with increased functional limitations that lead to needing assistance with personal care (i.e., toileting, bathing, grooming, dressing, or eating) and greater care needs, such as increased risk of falls or increased risk of infection (i.e., urinary-tract infection or pneumonia) because of lack of mobility, could possibly prevent or reduce inpatient hospital admission. Moreover, early identification of and intervention for comorbid conditions in the primary care setting could also reduce inpatient admissions among PWD. Special care should be given when patients with personal-care dependencies are transitioned from hospital to home, as inappropriate transitions of care can lead to readmission [25].

Unlike other studies [9, 21], this study did not find enabling factors, such as income, priority status, or need factors (i.e., depression [8] and other chronic conditions) other than personal-care dependency and behavioral problems, to predict utilization. This study found that the predisposing factor of having a spouse predicts inpatient utilization. This is possibly because of the spousal caregiver's close attention to the patient's condition and ability to bring the PWD to the hospital when the need arises. Caregivers of PWD often assist with activities of daily living, including toileting, bathing, and feeding [1], and, thus, are intimately aware of their loved one's care needs. Additionally, it is possible that frail PWD who would otherwise have been discharged from inpatient care to a subacute care facility are discharged home because of the presence of a caregiving spouse [26]. On the other hand, spousal caregivers of PWD are often older and more likely to have their own health problems and vulnerabilities, compared with younger, nonspousal caregivers (i.e., adult children). Depending on the degree of the caregiver's own health problems, inpatient hospitalization of the PWD may provide much-needed respite care. Nonetheless, further examination of inpatient utilization among PWD with spousal caregivers is needed.

This study has several limitations. Generalizability is limited because of the small sample size and because of the underrepresentation of women in a sample of veterans. However, the study sample was drawn from five different locations throughout the United States. This sample represents only those veteran and caregiver dyads that had complete data for 12 months and may, therefore, underreport the true number of hospitalizations, since veterans may have been admitted to a hospital, discharged to a skilled nursing facility or nursing home, and then dropped out of the study. Additionally, the nature of the PWD's hospitalization was not available to include in the analyses; this would have provided additional insight into the reasons for hospital admission. Finally, findings should be interpreted with caution, as this was a secondary data analysis that examined multiple predictors, which increases the likelihood of a type I error.

## 5. Conclusions

Despite its limitations, this study adds to the literature regarding the drivers of healthcare utilization among PWD. The finding that personal-care dependency leads to initial hospital admission as well as readmission highlights the need for appropriate levels of care for PWD as inappropriate transitions of care have been reported to lead to readmissions [25]. For example, PWD with little functional impairment are unlikely to thrive in a restrictive environment, such as a nursing home, but may instead need appropriate support in the community. However, individuals with increased care needs may experience decline, including possible hospitalization, if they are in an environment without enough support. Special care should be given to the appropriate environment for PWD with care dependencies. If it is appropriate for a PWD to stay in his/her own home, it is important to provide the caregiver with the resources necessary to safely keep the loved one at home; this can include increased training for the caregiver, in-home medical support, and/or home adaptations for the PWD. If a care setting other than the home would be better for the PWD, then there should be careful evaluation of the level of stimuli and level of care needed by the PWD prior to making the transition. Moreover, as new models of patient-centered medical homes for older adults are developed, careful attention to the needs of PWD and their caregivers is needed.

## Figures and Tables

**Table 1:** Descriptive statistics of demographic variables for persons with dementia and their caregivers.

	Descriptive stats	
	Inpatient admission	
	Yes (*n* = 104)	No (*n* = 192)
*Patient *		
Age (mean, std)	78.6 (8.0)	78.5 (8.1)
Gender		
Male (*n*, %)	102 (98)	188 (98)
Female (*n*, %)	2 (2)	4 (2)
Race		
White (*n*, %)	90 (87)	164 (85)
Other (*n*, %)	14 (13)	28 (15)
Education		
≤High school (*n*, %)	48 (46)	100 (52)
>High school (*n*, %)	56 (54)	92 (48)
Marital status		
Married (*n*, %)	87 (84)	142 (74)
Not married (*n*, %)	17 (16)	50 (26)
Income		
<$20,000 (*n*, %)	27 (26)	42 (22)
$20,000–$40,000 (*n*, %)	36 (35)	81 (42)
>$40,000 (*n*, %)	41 (39)	69 (36)
Priority score		
Priority 1	11 (10)	21 (11)
Priorities 2, 3, 4, 5, and 6	54 (52)	117 (61)
Priorities 7a, 7c, 8a, and 8c	39 (38)	54 (28)
Miles to VA (mean, std)	21.5 (34.3)	19.1 (18.2)
*Caregiver *		
Age (mean, std)	70.1 (11.6)	68.1 (12.3)
Gender		
Male (*n*, %)	4 (4)	7 (4)
Female (*n*, %)	100 (96)	185 (96)
Race		
White (*n*, %)	86 (83)	161 (84)
Other (*n*, %)	18 (17)	31 (16)
Education		
≤High school (*n*, %)	44 (42)	121 (63)
>High school (*n*, %)	60 (58)	71 (37)
Marital status		
Married (*n*, %)	93 (89)	166 (86)
Not married (*n*, %)	11 (11)	26 (14)
Family/friends who helped (mean, std)	4.9 (4.0)	4.6 (4.2)

VA: veterans administration.

**Table 2:** Number of veterans with inpatient hospital admissions over 12 months.

	Baseline		6 Months		12 Months		Total patients with an inpatient admission after baseline	
	*N* (%)	Mean (std)	*N* (%)	Mean (std)	*N* (%)	Mean (std)	*N* (%)	Mean (std)
Non-VA inpatient admission		2.56 (0.7)		2.36 (0.8)		3.00 (1.7)		2.93 (1.7)
Yes	47 (15.9)		44 (14.9)		45 (15.2)		74 (25.0)	
No	249 (84.1)		252 (85.1)		251 (84.8)		222 (75.0)	
VA inpatient admission		2.50 (0.8)		2.33 (0.6)		2.67 (0.6)		2.33 (0.7)
Yes	29 (9.8)		18 (6.1)		25 (8.5)		36 (12.2)	
No	267 (90.2)		278 (93.9)		271 (91.6)		260 (87.8)	
Both VA and non-VA Inpatient admission		2.53 (0.7)		2.53 (1.0)		2.87 (1.5)		2.53 (1.0)
Yes	2 (0.7)		2 (0.7)		1 (0.3)		6 (2.0)	
No	294 (99.3)		294 (99.3)		295 (99.7)		290 (98.0)	
Total inpatient admission*		2.53 (0.7)		2.53 (1.0)		2.87 (1.5)		2.84 (1.5)
Yes	74 (25.0)		60 (20.3)		69 (23.3)		104 (35.1)	
No	222 (75.0)		236 (79.7)		227 (76.7)		192 (64.9)	

*Total inpatient admission (yes) = “Non-VA inpatient admission (yes)” + “VA inpatient admission (yes)” − “Both VA and non-VA inpatient admission (yes)”; VA: veterans administration.

**Table 3:** Predisposing factors^∗^ for veterans' utilization of inpatient hospitalization.

	Univariate logistic regression (*n* = 296)			Univariate negative binomial (*n* = 104)		
	Estimate	*P* value	Confidence interval	Estimate	*P* value	Confidence interval
*Patient *						
Age	1.002	0.8998	0.972–1.032	0.0108	0.2804	−0.0088–0.0305
Gender	0.830	0.8316	0.148–4.641	−0.0839	0.8864	−1.2356–1.0677
Male						
Female^†^						
Race	0.970	0.9367	0.460–2.045	0.1696	0.4654	−0.2859–0.6251
White^†^						
Other						
Education	0.817	0.4130	0.503–1.326	0.0086	0.9546	−0.2865–0.3037
≤High school						
>High school^†^						
Marital status	0.502	0.0312	0.269–0.940	0.2169	0.2955	−0.1895–0.6233
Married^†^						
Not married						
*Caregiver *						
Age	1.014	0.1801	0.993–1.036	−0.0067	0.2959	−0.0192–0.0058
Gender	1.057	0.9314	0.299–3.731	−0.1214	0.7707	−0.9379–0.6951
Male						
Female^†^						
Race	1.183	0.6298	0.597–2.346	0.0194	0.9302	−0.4153–0.4542
White^†^						
Other						
Education	1.276	0.3367	0.776–2.009	0.0733	0.6377	−0.2317–0.3782
≤High school						
>High school^†^						
Marital status	0.734	0.4243	0.343–1.569	0.0665	0.7846	−0.4103–0.5433
Married^†^						
Not married						
Family/friends who helped	1.014	0.6482	0.956–1.075	−0.0143	0.4972	−0.0557–0.0271

*Controlled for site, ^†^reference category.

**Table 4:** Need factors^∗^ for veterans' utilization of inpatient hospitalization.

	Descriptive stats		Univariate logistic regression (*n* = 296)			Univariate negative binomial (*n* = 104)		
	Inpatient admission							
	Yes (*n* = 104)	No (*n* = 192)	Estimate	*P* value	Confidence interval	Estimate	*P* value	Confidence interval
*Patient *								
Personal care dependency (mean, std)	3.4 (3.5)	2.4 (3.0)	1.108	0.0074	1.028–1.194	0.0504	0.0139	0.0102–0.0906
Cognitive impairment composite (mean, std)	6.7 (3.8)	6.9 (3.6)	0.989	0.7436	0.926–1.056	0.0172	0.3898	−0.0220–0.0565
Behavior problems composite (mean, std)	2.8 (2.6)	2.5 (2.6)	1.063	0.2053	0.967–1.168	0.0525	0.0579	−0.0018–0.1067
Chronic conditions (mean, std)	4.6 (2.2)	4.6 (2.3)	1.033	0.5684	0.925–1.152	0.0377	0.2695	−0.0292–0.1047
*Caregiver *								
Depression (mean, std)	4.0 (3.4)	4.4 (3.4)	0.961	0.2828	0.894–1.033	0.0217	0.2979	−0.0192–0.0627
Role captivity composite (mean, std)	3.6 (1.4)	3.7 (1.5)	0.945	0.4990	0.802–1.114	0.0482	0.3705	−0.0573–0.1536
Relationship strain (mean, std)	7.2 (2.2)	7.0 (2.3)	1.026	0.6365	0.922–1.142	0.0189	0.5674	−0.0459–0.0838
Physical health strain (mean, std)	3.8 (1.5)	3.6 (1.4)	1.079	0.3726	0.913–1.274	0.0512	0.3023	−0.0460–0.1484

*Controlled for site.

**Table 5:** Enabling factors^∗^ for veterans' utilization of inpatient hospitalization.

	Univariate logistic regression (*n* = 296)			Univariate negative binomial (*n* = 104)		
	Estimate	*P* value	Confidence interval	Estimate	*P* value	Confidence interval
Income						
<$20,000	1.164	0.3706	0.622–2.181	−0.0998	0.5996	−0.4726–0.2729
$20,000–$40,000	0.811	0.2693	0.462–1.421	−0.0920	0.6074	−0.4432–0.2591
**>$40,000	—	—	—	—	—	—
Priority score						
**Priority 1	—	—	—	—	—	—
Priorities 2, 3, 4, 5, and 6	0.844	0.2312	0.376–1.892	−0.1497	0.5301	−0.6171–0.3177
Priorities 7a, 7c, 8a, and 8c	1.368	0.1822	0.583–3.211	0.0020	0.9935	−0.4855–0.4896
Miles to VA	1.004	0.3940	0.995–1.014	−0.0021	0.4523	−0.0074–0.0033

*Controlling for site, **reference variable.

**Table 6:** Multivariate analysis^∗^ of predictors of veterans' utilization of inpatient hospitalization (*n* = 195).

	Logistic regression (*n* = 296)			Negative binomial (*n* = 104)		
	Estimate	*P* value	Confidence interval	Estimate	*P* value	Confidence interval
*Patient *						
Marital status	0.493	0.029	0.261–0.930	—	—	—
Personal care dependency	1.048	0.007	1.029–1.197	0.040	0.035	0.003–0.085
Behavior problems	—	—	—	0.044	0.157	−0.015–0.095
*VA site *						
Houston versus Providence (1 versus 5)	1.048	0.4556	0.535–2.054	−0.152	0.487	−0.579–0.276
Boston versus Providence (2 versus 5)	0.542	0.2590	0.194–1.509	0.554	0.034	0.041–1.067
Oklahoma City versus Providence (3 versus 5)	0.519	0.2541	0.171–1.575	−0.445	0.309	−1.302–0.412
Beaumont versus Providence (4 versus 5)	1.575	0.0163	0.849–2.921	0.134	0.458	−0.220–0.487

*Controlled for site.
